# Survival Analysis of Risk Factors for Death in Patients With TB Based on Clinical and Imaging Characteristics

**DOI:** 10.1155/carj/1050817

**Published:** 2026-07-08

**Authors:** Jing Han, Peng Li, Lixia Shi, Yi Xie, Xiaolong Liu, Wanjie Yang, Zhiheng Xing

**Affiliations:** ^1^ Department of Medical Affairs, Haihe Hospital, Tianjin University, Tianjin, 300350, China, tju.edu.cn; ^2^ Tianjin Institute of Respiratory Diseases, Tianjin, 300350, China, tju.edu.cn; ^3^ TCM Key Research Laboratory for Infectious Disease Prevention for State Administration of Traditional Chinese Medicine, Tianjin, China; ^4^ Haihe Clinical School, Tianjin Medical University, Tianjin, 300350, China, tijmu.edu.cn; ^5^ Department of Radiology, Haibin People’s Hospital of Binhai New Area, Tianjin, 300280, China; ^6^ Department of Respiratory and Critical Care Medicine, Haihe Hospital, Tianjin University, Tianjin, 300350, China, tju.edu.cn; ^7^ Department of Science and Education, Haihe Hospital, Tianjin University, Tianjin, 300350, China, tju.edu.cn; ^8^ Department of Information, Haihe Hospital, Tianjin University, Tianjin, 300350, China, tju.edu.cn; ^9^ Department of Critical Care Medicine, Haihe Hospital, Tianjin University, Tianjin, 300350, China, tju.edu.cn; ^10^ Department of Radiology, Haihe Hospital, Tianjin University, Tianjin, 300350, China, tju.edu.cn

**Keywords:** DOTS, HIV, nomogram, tuberculosis

## Abstract

**Background:**

This study was performed to explore the predictive factors for mortality among patients with tuberculosis undergoing directly observed treatment, short‐course (DOTS) therapy and to develop a clinically applicable visualization tool for mortality risk prediction.

**Methods:**

We conducted a retrospective cohort study of 9270 patients (8884 survivors and 386 deaths, yielding a mortality rate of 4.16%) from 2014 to 2024, utilizing data from the tuberculosis management information system in Tianjin. Cox proportional hazards regression was used to identify independent risk factors, a nomogram model was constructed, and model performance was evaluated using cross‐validation and ROC curves.

**Results:**

Male sex, advanced age, human immunodeficiency virus–positive status, pulmonary cavities, initial sputum positivity, pericardial effusion, and miliary nodules emerged as independent risk factors for DOTS mortality. The nomogram demonstrated an area under the curve of 0.81 for predictions at 2, 6, and 12 months; calibration curves revealed high concordance between predicted and actual risk (with average absolute errors of 0.001, 0.001, and 0.004, respectively); receiver operating characteristic curves confirmed robust discriminative ability of the model.

**Conclusion:**

The nomogram developed in this study successfully integrates multiple mortality predictive factors and exhibits exhibiting excellent discriminatory power and calibration performance, thereby providing quantitative decision support for early identification and personalized intervention strategies in high‐risk patients with tuberculosis receiving DOTS therapy.

## 1. Introduction

Tuberculosis (TB), one of the top 10 causes of death globally, continues to pose a significant threat to human health. According to data from the World Health Organization (WHO), by 2023, there will be 10.8 million new cases of TB worldwide, resulting in 1.25 million deaths; thus, TB will become the leading cause of mortality from infectious diseases globally [1]. Although standardized treatment regimens such as the combination of rifampicin and isoniazid have significantly reduced the mortality rate of active TB, patient outcomes are still influenced by multiple complex factors, including host immune status [2], drug resistance [3], comorbidities [4], and delayed diagnosis and treatment [5].

Previous studies on the prognostic factors of patients with TB have primarily focused on individual health conditions, such as age, sex, and underlying diseases, or on the severity of the disease, including cavity formation and drug resistance [5–7]. However, TB is a complex clinical condition that often results from an interplay of multiple factors. The current research lacks a clear and in‐depth understanding of the interactions between these factors and their weight distributions in different clinical settings.

TB poses diverse clinical manifestations, encompassing cavities, pericardial effusion, and miliary nodules, with a complex underlying pathogenesis. The interactions among these factors and the utilization of imaging data for prognostic prediction warrant further investigation. This study sought to systematically analyze independent risk factors for adverse outcomes in patients with TB during the DOTS period. Through integration of clinical characteristics, imaging data, and epidemiological information, we aimed to establish a scientific foundation for personalized treatment approaches and precision interventions, ultimately enhancing patient outcomes, treatment efficacy, and quality of life.

## 2. Materials and Methods

### 2.1. Research Object

This single‐center retrospective cohort study enrolled patients diagnosed with pulmonary TB who initiated anti‐TB treatment at designated TB hospitals in Tianjin between January 2014 and April 2024. The inclusion criteria comprised (1) active pulmonary TB confirmed by pathogen detection or clinical diagnosis in accordance with the Tuberculosis Diagnostic Criteria (WS288‐2008/2017) [8, 9] and the Guidelines for the Diagnosis and Treatment of Tuberculosis issued by the Chinese Society for Tuberculosis, Chinese Medical Association, (2) age 18 years or older, and (3) receipt of standard anti‐TB treatment. Exclusion criteria encompassed (1) extra pulmonary TB and (2) absence of > 20% data during the treatment period. Among 13,896 registered individuals, 384 individuals under 18 years of age and 4242 patients with > 20% missing data during the treatment period were excluded. The Tianjin Haihe Hospital Ethics Committee approved this study (Approval No. 2024HHKT‐002). Patients’ personal information was anonymized, and the requirement for informed consent was waived, given the retrospective nature of the investigation.

### 2.2. Data Sources

This retrospective cohort investigation utilized the TB management information system of the hospital to extract the demographic data and medical records of the patients. Demographic information encompassed sex and age, while medical records comprised follow‐up status, human immunodeficiency virus (HIV) status, initial sputum culture results, drug resistance patterns, delays in seeking medical care and diagnosis, clinical symptoms, imaging findings (including cavities, consolidation, bronchiectasis with thickening, narrowing, distortion, lymphadenopathy, linear shadows, pericardial effusion, and miliary nodules), treatment duration, and outcomes. For imaging feature extraction, structured report texts from imaging examinations performed at our institution were retrieved from the electronic medical records of the patients. Two researchers independently extracted key lesion features in a blinded manner. When multiple examination reports existed for a single patient, only the initial report was incorporated into the analysis. Outcomes—encompassing death, cure, treatment completion, failure, loss to follow‐up, and other outcomes—were defined according to the “Guidelines for the Implementation of the Chinese Tuberculosis Prevention and Control Plan” [10]. A patient delay was defined as exceeding 14 days from symptom onset to medical consultation, while diagnostic delay was defined as exceeding 14 days from medical consultation to diagnosis [11]. In Tianjin, patients with confirmed TB undergo a comprehensive clinical assessment and promptly initiate anti‐TB treatment on the day of diagnosis. Consequently, the date of treatment initiation was used as the time of origin for survival analysis, which aligned with the date of diagnosis.

### 2.3. Treatment Principles

The requirements of the guidelines for the supervision of the treatment period are as follows. (1) Patients with newly diagnosed pulmonary TB are generally supervised for 6 months; if the sputum smear test results of newly smear‐positive pulmonary TB patients remained positive at the end of 2 months, the intensive treatment is extended for 1 month, and the treatment is supervised for 7 months. (2) Patients with retreated pulmonary TB were generally supervised for 8 months; those who could not use streptomycin were provided 1 month of intensive treatment and 9 months of supervised treatment. If the retreated smear‐positive pulmonary TB patients remained positive for sputum bacteria at the end of the second month after treatment, patients treated with the streptomycin regimen should extend the retreatment and intensive treatment for 1 month, with supervised treatment for 9 months, while patients who did not use the streptomycin regimen extend the intensive period of treatment for another month, with the treatment plan unchanged in the continuous period and the supervised treatment for 10 months [5].

The investigation used a survival analysis framework, with TB initiation of anti‐TB treatment serving as the starting event. Death during follow‐up represented the endpoint event, while censored events included treatment course completion, cure achievement, loss to follow‐up, treatment failure, and voluntary withdrawal. The follow‐up period concluded on April 1, 2025.

### 2.4. Statistical Processing

Patient medical records were compiled in an Excel database, and statistical analyses were performed using SPSS 25.0 and *R* software (R4.4.3). The life table method was used to estimate cumulative survival rates. Intergroup survival rates were compared using the log‐rank test, while Cox proportional hazards regression was used to explore factors influencing patient mortality during treatment. Survival time and survival outcome (death) served as dependent variables, while factors demonstrating significance at *p* < 0.05 in the univariate analysis and those possessing practical significance (such as age) were selected as independent variables for the Cox proportional hazards model.

Independent risk factors were imported into *R* software (R 4.4.3), and a prediction model nomogram was constructed using the RMS package. Internal validation of the nomogram model used 10‐fold cross‐validation with 1000 iterations. Receiver operating characteristic (ROC) curves for the mortality risk prediction nomogram were generated, with area under the curve (AUC) and Brier scores calculated to assess model calibration. Alpha was set at 0.05 (two‐sided), with each line segment score of the variable calculated based on the regression coefficient (*β*) and range (distance).

## 3. Results

### 3.1. Baseline Data

Overall, 9270 patients received standardized anti‐TB treatment and were included in the evaluation of anti‐TB efficacy. Among them, 6430 (69.35%) and 2840 (30.65%) were male and female, respectively; moreover, their average age was 49.68 ± 19.59 years (ranging from 18 to 96 years). Here, HIV‐positive status, positive sputum culture at first visit, drug resistance, delayed diagnosis, delayed confirmation, and hemoptysis were observed in 16 (0.17%), 5,506, 1221 (13.17%), 2778 (29.97%), 4876 (52.60%), and 192 (2.07%) patients, respectively. Regarding imaging findings, cavities, consolidation, bronchial lesions, enlarged lymph nodes, linear shadows, pericardial effusions, and miliary nodules were observed in 4123 (44.48%), 1983 (21.39%), 736 (7.94%), 484 (5.22%), 892 (9.62%), 356 (3.84%), and 59 (0.64%) patients, respectively.

A total of 386 (4.16%) patients died, while 8884 (95.84%) survived. Moreover, 51.03% (197/386) died within the first 2 months of treatment (Table 1). The median treatment duration of surviving patients was 6.2 months (interquartile range [IQR] 5.8–6.8), while that of deceased patients was significantly shorter (*p* < 0.001) at 2.8 months (IQR 1.2–4.5).

**TABLE 1:** Survival probabilities throughout the course of treatment in 9270 patients with TB.

Survival time/months	Initial number of cases	Died	Number of deletions during the period	Cumulative survival analysis at the end of the period
0∼	9270	119	143	98.71
1∼	9008	78	74	97.85
2∼	8856	59	77	97.19
3∼	8720	35	42	96.80
4∼	8643	22	65	96.56
5∼	8556	31	517	96.19
6∼	8008	18	4403	95.90
7∼	3587	13	931	95.50
8∼	2643	2	1255	95.40
9∼	1386	6	324	94.93
10∼	1056	2	141	94.74
11∼	913	1	98	94.63
12∼	814	0	814	94.63

### 3.2. Disease‐Specific Deaths During DOTS Among Patients With TB

A comparison of the log‐rank test results showed that there were statistically significant differences in mortality rates during the DOTS period among patients with pulmonary TB, including sex, age, HIV test results, initial sputum culture results, patient delay, and imaging signs such as cavities, consolidation, bronchial lesions, enlarged lymph nodes, pericardial effusion, and miliary nodules (*p* < 0.05) (Table 2).

**TABLE 2:** Univariate analysis of risk factors for death during the treatment period in 9270 patients with TB.

Variable		Total cases	State		HR	95%CI		*P*
			Alive	Died		Lower	Upper	
Sex	Female	2840	2774 (97.68%)	66 (2.32%)				
	Male	6430	6110 (95.02%)	320 (4.98%)	2.18	1.67	2.84	< 0.001

Age	< 60	6133	6037 (98.43%)	96 (1.57%)				
	≥ 60	3137	2847 (90.76%)	290 (9.24%)	6.32	4.99	8.02	< 0.001

HIV detection result	Negative/undetectable	9254	8871 (95.86%)	383 (4.14%)				
	Positive	16	13 (81.25%)	3 (18.75%)	4.58	1.47	14.28	0.004

Initial sputum culture results	Negative	3764	3676 (97.66%)	88 (2.34%)				
	Positive	5506	5208 (94.59%)	298 (5.41%)	2.38	1.88	3.02	< 0.001

Drug resistance	No	8049	7706 (95.74%)	343 (4.26%)				
	Yes	1221	1178 (96.48%)	43 (3.52%)	0.98	0.72	1.35	0.908

Treatment classification	New	7595	7316 (96.33%)	279 (3.67%)				
	Relapse	1675	1568 (93.61%)	107 (6.39%)	1.65	1.32	2.07	< 0.001

Patient delay	No	6492	6242 (96.15%)	250 (3.85%)				
	Yes	2778	2642 (95.10%)	136 (4.90%)	1.27	1.03	1.57	0.024

Diagnostic delay	No	4394	4203 (95.65%)	191 (4.35%)				
	Yes	4876	4681 (96.00%)	195 (4.00%)	0.92	0.75	1.12	0.41

Hemoptysis	No	9078	8704 (95.88%)	374 (4.12%)				
	Yes	192	180 (93.75%)	12 (6.25%)	1.50	0.84	2.66	0.166

Cavity	No	5147	4967 (96.50%)	180 (3.50%)				
	Yes	4123	3917 (95.00%)	206 (5.00%)	1.47	1.20	1.79	< 0.001

Consolidation	No	7287	7027 (96.43%)	260 (3.57%)				
	Yes	1983	1857 (93.65%)	126 (6.35%)	1.79	1.45	2.21	< 0.001

The bronchi are dilated, thickened, narrowed, and twisted	No	8534	8193 (96.00%)	341 (4.00%)				
	Yes	736	691 (93.89%)	45 (6.11%)	1.55	1.14	2.12	0.005

The lymph nodes are enlarged	No	8786	8436 (96.02%)	350 (3.98%)				
	Yes	484	448 (92.56%)	36 (7.44%)	1.88	1.33	2.64	< 0.001

A thread of shadow	No	8378	8022 (95.75%)	356 (4.25%)				
	Yes	892	862 (96.64%)	30 (3.36%)	0.78	0.54	1.13	0.19

Pericardial effusion	No	8914	8569 (96.13%)	345 (3.87%)				
	Yes	356	315 (88.48%)	41 (11.52%)	3.05	2.21	4.22	< 0.001

Miliary nodules	No	9211	8835 (95.92%)	376 (4.08%)				
	Yes	59	49 (83.05%)	10 (16.95%)	4.35	2.32	8.15	< 0.001

### 3.3. Multivariate Analysis of the Effects of Death During DOTS on Patients With TB

The 12 indicators that showed statistically significant differences, including sex; age; HIV test results; treatment classification; patient delay; initial sputum culture results; cavities; pericardial effusion; miliary nodules;consolidation; bronchial dilation, thickening, or twisting; and enlarged lymph nodes; were included in the Cox proportional risk regression analysis. The results indicated that male sex, advanced age, HIV positivity, cavities, positive first sputum culture results, pericardial effusion, and miliary nodules were risk factors for mortality during DOTS (*p* < 0.05) (Table 3).

**TABLE 3:** Multivariate Cox proportional risk regression analysis of the influencing factors of death during antituberculosis treatment in patients with pulmonary TB.

		**β**	**SE**	**Wald** **χ** ^2^	**p**	**HR**	**95%CI**	

Age		0.06	0.00	280.89	< 0.001	1.06	1.05	1.07

Sex	Female							
	Male	0.65	0.14	22.46	< 0.001	1.91	1.46	2.50

HIV detection result	Negative/not measured							
	HIV positive	1.70	0.58	8.55	0.003	5.48	1.75	17.12

First sputum results	Negative/not measured							
	Positive	0.54	0.13	19.03	< 0.001	1.72	1.35	2.20

Cavities	No							
	Yes	0.24	0.11	5.20	0.023	1.27	1.03	1.57

Pericardial effusion	No							
	Yes	0.47	0.17	7.85	0.005	1.60	1.15	2.22

Miliary nodules	No							
	Yes	1.26	0.32	15.31	< 0.001	3.51	1.87	6.59

### 3.4. Mortality Risk Prediction Line Graph

**FIGURE 1:**
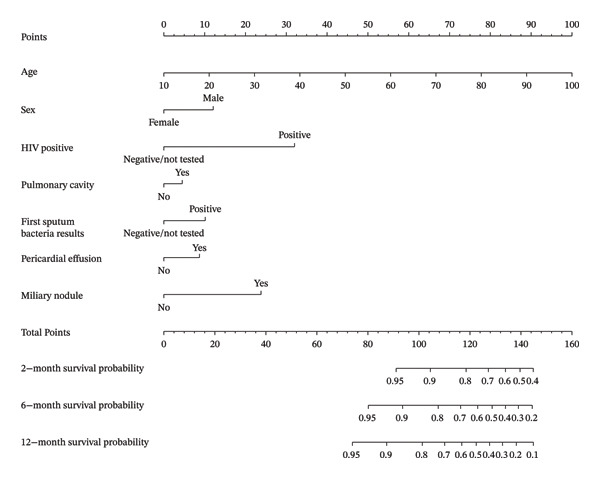
Nomogram used to predict the risk of death in patients with tuberculosis (TB) using the DOTS strategy.

The impact of the predicted indicators on the outcomes was integrated into a Cox regression model. Scaled line segments are plotted on the same plane at a specific ratio to illustrate the relationships among the variables in the prediction model. The scores for each condition were aggregated, and the total score was calculated by summing the scores. Finally, the predicted survival time was determined using the total score (Figure 1 and Table 4).

**TABLE 4:** Nomogram score of risk of death in patients with pulmonary TB during the DOTS period.

Variables	Nomogram scores
Age	1.1/year
Sex	12.1/male
HIV	32.0/positive
Pulmonary cavity	4.5/yes
First sputum bacteria results	10.1/positive
Pericardial effusion	8.7/yes
Miliary nodules	23.8/yes

### 3.5. Validation of the Nomogram

**FIGURE 2:**
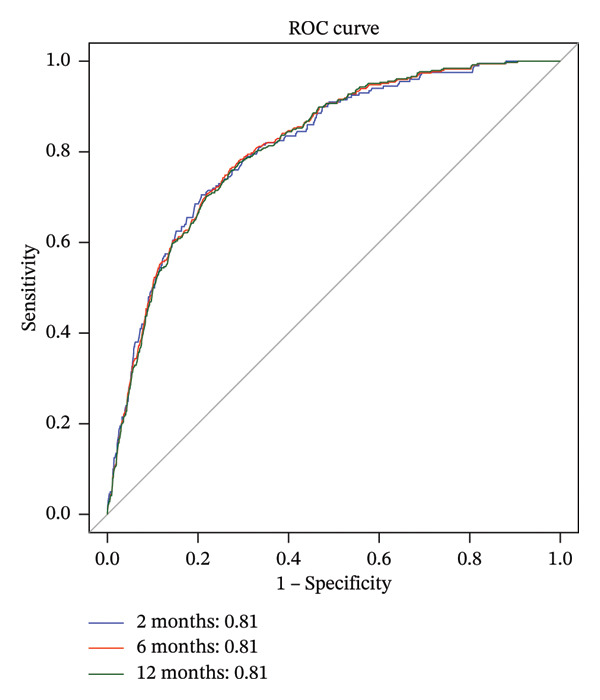
The ROC curve and AUC of the nomogram for the risk of mortality at 2, 6, and 12 months.

Figure 1 displays a comprehensive predictive model that integrates all factors influencing the mortality rates of patients with TB. The average absolute errors between predicted and actual death probabilities were 0.001 at both 2 and 6 months and 0.004 at 12 months, indicating excellent calibration performance. Overall discrimination yielded a Harrell’s C‐index of 0.80 (standard error [SE] = 0.03). ROC curves generated using *R* software (Figure 2) demonstrated AUC values at critical time points (2, 6, and 12 months) of 0.81 (SE = 0.02), 0.81 (SE = 0.01), and 0.81 (SE = 0.02), respectively, all exceeding 0.81 and reflecting superior discriminative capacity. Additionally, relatively low Brier scores (2 months, 0.02; 6 months, 0.03; 12 months, 0.05) further validated the overall accuracy of the model in predicting mortality probabilities across different time intervals.

## 4. Discussion

Through multivariate Cox regression analysis, we identified male sex, advanced age, HIV positivity, cavity formation, initial positive sputum culture, pericardial effusion, and miliary nodules as independent risk factors for DOTS‐period mortality. While our previous investigations have contributed epidemiological data regarding these patients [5, 11], the current study advances this knowledge by integrating clinical manifestations, imaging markers (including cavities and miliary nodules), and epidemiological data to construct a comprehensive multidimensional risk‐warning model. These findings enhance our understanding of TB‐related mortality mechanisms and provide precise targets for personalized treatment strategies, including intensified interventions for patients with pulmonary cavities and immunomodulatory approaches for those with HIV coinfection.

The median treatment time for deceased patients (2.8 months, IQR 1.2–4.5) was significantly shorter than that for survivors (6.2 months, IQR 5.8–6.8; *p* < 0.001). This observation can be mainly attributed to premature termination of treatment due to death. During treatment, survivors may experience treatment deviations due to personalized plans targeting adverse drug reactions, comorbidities, or disease severity. Our investigation demonstrates that age represents a critical risk factor for TB mortality, with each additional year of age conferring a 1.057‐fold increase in death risk. These findings align with previous studies from Malaysia [12], Ethiopia [13], and Iran [14], which reported age‐specific hazard ratios (HRs) ranging from 1.03 to 1.10. Progressive age‐related decline in pulmonary function, coupled with diminished immunity and nutritional status, heightens susceptibility to adverse TB outcomes [15, 16]. Furthermore, older patients with TB frequently experience severe or recurrent chemotherapy‐related adverse effects and protracted disease courses, potentially compromising adherence to anti‐TB drug regimens [17]. Our analysis revealed significantly higher mortality risk among male patients compared to that of females (HR = 1.798), which is consistent with a South African cohort study [18]. This may be attributed to delayed healthcare‐seeking behavior and smoking exposure. Female patients with TB typically demonstrate superior health protection awareness and treatment adherence compared to males. The combination of elevated occupational and lifestyle stressors, increased labor intensity, and higher prevalence of smoking and alcohol consumption among males often results in diminished health attention, leading to compromised treatment adherence and elevated mortality risk from irregular treatment [11].

Our findings indicate that patients who are HIV‐positive experience a 5.02‐fold higher risk of adverse outcomes compared to patients who are HIV‐negative. This observation aligns with the findings of investigations from Ethiopia [19, 20], Somalia [21], South Africa [22], Georgia [23], and Brazil [24], which consistently demonstrated superior treatment success rates among patients with HIV‐negative TB compared to those of individuals with TB/HIV coinfection. In coinfected patients, HIV systematically compromises the immune system, particularly targeting CD4+ T lymphocytes, resulting in profound immunosuppression. This severe immune dysfunction impairs the capacity of the patients to effectively control *Mycobacterium tuberculosis* infection and dissemination, precipitating rapid clinical deterioration [25]. Consequently, enhancing detection and diagnostic capabilities for both acquired immunodeficiency syndrome and TB, while implementing comprehensive preventive and control measures for coinfected individuals, remains paramount.

Our investigation additionally revealed that patients with positive initial pathogen tests for pulmonary TB demonstrated a 1.767‐fold increased mortality risk compared to those with negative or untested results. This finding suggests that patients with positive initial tests face an elevated risk of subsequent adverse outcomes [26]. An initial positive sputum culture serves as a crucial indicator of TB activity and transmissibility. Evidence indicates that lung imaging revealing cavities, consolidation, bronchiectasis, upper lobe involvement, multilobar disease, and lymphadenopathy significantly correlates with positive smear results [27]. Consequently, these patients necessitate intensive monitoring and enhanced treatment protocols.

From an imaging perspective, our analysis identified cavities, pericardial effusion, and miliary nodules as significant mortality risk factors during DOTS treatment. Evidence establishes the cavity presence as a critical indicator of TB progression and an independent predictor of poor prognosis [28, 29]. Cavitary TB formation mechanisms primarily involve caseous necrotic tissue liquefaction and its subsequent expulsion via the respiratory tract, frequently accompanied by varying degrees of consolidation [29]. Clinical characteristics include a protracted disease course, prolonged sputum positivity, propensity for concurrent bronchial TB, heightened infectivity, resistance to cavity closure, suboptimal treatment outcomes, and elevated recurrence rates, collectively depleting physical reserves of the patients and increasing mortality risk. Cavities facilitate intrapulmonary *M. tuberculosis* dissemination, accelerate disease progression, cause extensive pulmonary tissue destruction, and compromise lung function [28]. Urbanowski et al. demonstrated that the presence of a radiological cavity within the initial two treatment months is correlated with an elevated risk of treatment failure and recurrence. Persistent cavities at 6 months posttreatment initiation double the recurrence risk compared to that of patients achieving cavity closure following treatment completion [30]. Pericardial effusion potentially precipitates severe complications, including pericardial tamponade, arrhythmias, heart failure, and sudden cardiac death [31]. *M. tuberculosis* can disseminate to the pericardium through lymphatic or hematogenous routes, initiating inflammatory responses. Additionally, the organism may directly invade pericardial tissue from hilar or mediastinal lymph nodes via lymphatic channels, inducing localized inflammation [32]. Given the substantial mortality associated with tuberculous pericarditis, prompt diagnosis and treatment remain essential. Miliary TB carries considerable morbidity and mortality burdens, with mortality rates approximating 15%–20% in pediatric populations and 25%–30% in adults [33–35]. This form results from extensive lymphohematogenous *M. tuberculosis* dissemination, characterized by distinctive small nodules resembling millet seeds on gross pathological examination [36]. Radiologically, miliary pulmonary TB manifests as bilateral diffuse miliary nodular infiltrates, representing a pathognomonic imaging finding. Hematogenous mycobacterial spread produces widespread visceral nodules, typically measuring 1‐2 mm with frequent caseous features. These nodules may remain radiographically undetectable on conventional chest radiography, contributing to diagnostic delays or missed diagnoses with potentially fatal consequences [37]. Patient delay and diagnostic delay represent two distinct dimensions of TB‐associated delay, with their impacts varying considerably across healthcare settings. Patient delay is consistently linked to disease progression and elevated mortality risk, while the prognostic effect of diagnostic delay is context‐dependent [38, 39]. In regions with well‐established TB control and prevention systems (e.g., Tianjin), the timely initiation of treatment after diagnosis effectively mitigates the impact of diagnostic delay, making its independent effect on mortality insufficient to reach statistical significance in the multivariate model. This finding highlights the importance of an efficient TB control system in offsetting the adverse effects of diagnostic delay.

We identified male sex, advanced age, HIV positivity, cavity formation, initial positive sputum culture, pericardial effusion, and miliary nodules as independent risk factors for DOTS‐period mortality. In this study, a comprehensive multidimensional risk nomogram model was constructed by integrating clinical manifestations, imaging biomarkers (including cavities and miliary nodules), and epidemiological data. Limitations of the study: First, as a single‐center study, the present research is inherently subject to selection bias, and caution should therefore be exercised when extrapolating the study findings to other regions or healthcare systems with different practice patterns. Second, owing to the structural constraints of the TB management information system, complete data on key comorbidities including diabetes mellitus were unavailable and could not be included in our analysis. Given that diabetes mellitus is a well‐recognized risk factor for TB‐related mortality, the absence of such data may have introduced residual confounding, which could lead to underestimation of the impact of comorbidities on clinical prognosis in our predictive model. Third, while drug resistance data were collected, the long study duration and lack of standardized data recording protocols during the early stages of the study period meant that only binary classification (resistant/susceptible) was possible. This precluded further stratification of specific drug resistance patterns, such as monoresistance, polydrug resistance, and multidrug resistance. In future investigations, we intend to conduct a multicenter prospective cohort study to validate the performance of the developed model in an independent external cohort.

## 5. Conclusions

The comprehensive multidimensional mortality risk assessment model developed through this investigation incorporates host factors (male sex and advanced age), coinfection status (HIV positivity), microbiological parameters (initial sputum culture positivity), and radiological findings. These components collectively constitute an integrated early warning system for all‐cause mortality during DOTS treatment. This model facilitates the timely identification of high‐risk individuals, enables population stratification, and supports the integration of the findings into clinical decision‐making frameworks. Implementation promotes systematic screening for high‐risk factors, facilitates targeted interventions for vulnerable patients, and ultimately optimizes DOTS treatment outcomes.

## Author Contributions

Study conception and design were performed by Jing Han, Peng Li, and Zhiheng Xing. Administrative support was provided by Jing Han and Wanjie Yang. Study materials or patient resources were managed by Wanjie Yang, Lixia Shi, Yi Xie, and Zhiheng Xing. Data collection and assembly were conducted by Jing Han, Lixia Shi, Xiaolong Liu, and Yi Xie. Data analysis and interpretation were carried out by Jing Han, Peng Li, Lixia Shi, Yi Xie, and Wanjie Yang. All authors participated in manuscript writing. Jing Han, Peng Li, Lixia Shi, Yi Xie, and Xiaolong Liu contributed equally to this work.

## Funding

This work was supported by the Tianjin Science and Technology Major Project (to W.J.Y.) (grant number 24ZXKJGX00070), the Tianjin Natural Science Foundation Project (to W.J.Y.) (grant number 23JCZDJC00970), the Tianjin Natural Science Foundation Project (to J.H.) (grant number 23JCQNJC01490), and the Tianjin Health Research Project (to W.J.Y.) (grant number TJWJ2024ZD009).

## Disclosure

All authors approved the final version of the manuscript.

## Conflicts of Interest

The authors declare no conflicts of interest.

## Data Availability

The data that support the findings of this study are available from the corresponding authors upon reasonable request.
